# RPGRIP1L helps to establish the ciliary gate for entry of proteins

**DOI:** 10.1242/jcs.220905

**Published:** 2018-10-26

**Authors:** Huawen Lin, Suyang Guo, Susan K. Dutcher

**Affiliations:** Department of Genetics, Washington University in St. Louis, St. Louis, MO 63110, USA

**Keywords:** Transition zone, Cilia, Basal body, *Chlamydomonas reinhardtii*

## Abstract

Mutations in transition zone genes change the composition of the ciliary proteome. We isolated new mutations in *RPGRIP1L* (denotated as *RPG1* in algae) that affect the localization of the transition zone protein NPHP4 in the model organism *Chlamydomonas reinhardtii*. NPHP4 localization is not affected in multiple new intraflagellar transport (IFT) mutants. We compared the proteome of cilia from wild-type and mutants that affect the transition zone (*RPGRIP1L*) or IFT (*IFT172* and *DHC1b*) by mass spectrometry. The *rpg1-1* mutant cilia show the most dramatic increase in cytoplasmic proteins. These nonciliary proteins function in translation, membrane remodeling, ATP production and as chaperonins. These proteins are excluded in isolated cilia from *fla11-1* (IFT172) and *fla24-1* (DHC1b). Our data support the idea that RPGRIP1L, but not IFT proteins, acts as part of the gate for cytoplasmic proteins. The *rpg1-1* cilia lack only a few proteins, which suggests that RPGRIP1L only has a minor role of in the retention of ciliary proteins. The *fla11-1* mutant shows the greatest loss/reduction of proteins, and one-third of these proteins have a transmembrane domain. Hence, IFT172 may play a role in the retention of proteins.

## Abstract (precis)

**Summary:** RPGRIP1L, a key protein of the transition zone that is needed for NPHP4 localization, helps to exclude cytoplasmic proteins from the cilia in *Chlamydomonas reinhardtii*.

## INTRODUCTION

Cilia are membrane-bound projections extending from the plasma membrane of most eukaryotic cells. Motile cilia move fluids over their surface. Primary cilia serve as antenna to receive environmental signals and respond via a large number of signaling pathways. Cilia also send signals via ectosomes (Wood et al., 2013; Long et al., 2016). Many of these signaling pathways are important for development and homeostasis. Defects in cilia cause a group of diseases called ciliopathies, and result in obesity, cystic kidney, blindness, skeletal malformations and nervous system abnormalities, as well as respiratory and laterality defects (Reiter and Leroux, 2017).

The basal body of cilia docks at the plasma membrane, permanently in many unicellular organisms or transiently in most metazoan tissues. The transition zone, which is recognized by the change from triplet to doublet microtubules, assembles at the distal end of the basal body. The transition fibers in *Chlamydomonas* or the distal and subdistal appendages in mammalian cells are attached to the microtubules in the transition zone (Fisch and Dupuis-Williams, 2011). The transition fibers may also serve as a docking site for intraflagellar transport (IFT) proteins (Deane et al., 2001). At the distal end of the transition zone are structures called Y-linkers (reviewed in Fisch and Dupuis-Williams, 2011) and they appear to anchor the doublet microtubules to the ciliary membrane. Several organisms, including *Giardia* and cycads, lack Y-linkers (Barker et al., 2014). The transition zone has three protein complexes called the MKS, NPHP and CEP290 modules (see Table S1 for gene names in human, *Caenorhabditis elegans* and *Chlamydomonas reinhardtii*) (Williams et al., 2011; Schouteden et al., 2015). These proteins are not essential for ciliary assembly in *C. elegans* since triple mutants that lack all the modules still assemble cilia (Schouteden et al., 2015). In many studies, these proteins localize to the transition zone (Fliegauf et al., 2006; Garcia-Gonzalo et al., 2011; Reiter et al., 2012; Williams et al., 2011). Mutations in these genes result in a constellation of clinical symptoms that include retinal, kidney, and neurodevelopmental defects as well as early lethality (Czarnecki and Shah, 2012). RPGRIP1L (denoted as *RPG1* in algae) has been implicated by genetic studies in *C. elegans* as a key player in the transition zone that interacts with both the MKS and NPHP proteins (Williams et al., 2011; Schouteden et al., 2015), and it is required for TMEM237 localization (Huang et al., 2011). Homologs of these genes are found in the *Chlamydomonas* genome and in the transition zone proteome (Diener et al., 2015). BioID/proximity mapping identified 66 proteins that interact with RPGRIP1L; 11 of these are also found using FLAG-tagged protein for immunoprecipitation. These studies find that RPGRIP1L clusters with both transition zone and centrosomal appendage proteins (Gupta et al., 2015). Mouse studies link the dosage of RPGRIP1L with appetite control (Stratigopoulos et al., 2014). RPGRIP1L appears to be a central protein of the transition zone.

The function of the transition zone as a ciliary gate has been probed in both mutants and via siRNA. In *Chlamydomonas*, mutations in either *CEP290* (Craige et al., 2010) or *NPHP4* (Awata et al., 2014) result in the presence of a small number of inappropriate proteins in cilia and the absence of some proteins. In mouse embryos and hTERT-RPE1 cells, tectonic-1 (TCTN1) as well as TCTN2 and CC2DC2A, which are part of the MKS module, are needed to localize the membrane-associated proteins ARL13a, adenylate cyclase III (AC3), Smoothened and polycystin-2 (PKD2) to the primary cilium (Garcia-Gonzalo et al., 2011). Knockdown of TMEM67 affects AC3 localization but not that of the other proteins (Garcia-Gonzalo et al., 2011). In *C. elegans*, loss of transition zone proteins in any of the three modules causes RPI-2 (the retinitis pigmentosa 2 homolog), and the transmembrane proteins TRAM-1a and TMEM67 to accumulate in the cilia instead of the transition zone (Williams et al., 2011; Li et al., 2016). The loss of RPGRIP1L/MKS5 in *C. elegans* suggests that it plays a role in creating a membrane diffusion barrier (Jensen et al., 2015). These transition zone proteins clearly play a role in the entry of ciliary proteins as well as the exclusion of cytoplasmic proteins. In addition, the ciliary gate has been proposed to behave in a similar manner to the nuclear pore (Takao et al., 2014). Endicott and Brueckner, by using siRNA, have shown that reducing the amount of the nuclear pore protein NUP98 limits the diffusion of proteins greater than 70 kDa into the cilium. This protein is important for import of the tubulin dimer (Endicott and Brueckner, 2018).

Ciliary assembly is dependent on the intraflagellar transport (IFT) system composed of at least 22 proteins (Taschner and Lorentzen, 2016). In *Chlamydomonas*, IFT proteins have been biochemically separated into two complexes called IFT-A, which plays a role in retrograde movement from the ciliary tip to the cell body, and IFT-B, which plays a role in anterograde movement (Piperno and Mead, 1997; Piperno et al., 1998; Cole et al., 1998; Iomini et al., 2001, 2009). A conditional mutation in IFT172, which is part of the IFT-B complex (Pedersen et al., 2005), shows a defective retrograde transport phenotype at the permissive temperature (Iomini et al., 2001). It has been postulated that IFT172 plays a role in IFT remodeling at the distal tip (Pedersen et al., 2005), thereby influencing retrograde IFT.

Here, we undertook an unbiased forward genetic screen for *Chlamydomonas* strains with ciliary assembly defects in order to identify new mutants that may affect basal body, transition zone or ciliary assembly and function. We identified 250 mutant strains. Whole-genome sequencing of a subset of these strains reveals mutations in the transition zone protein RPGRIP1L and in several IFT genes. Comparison of the protein composition of cilia from IFT mutants and wild-type suggests that IFT is not required to establish the ciliary gate that keeps cytoplasmic proteins out. As observed in other organisms, the gate appears to require components of the transition zone.

## RESULTS

### Isolation of mutants lacking NPHP4 in the transition zone

**Fig. 1.**
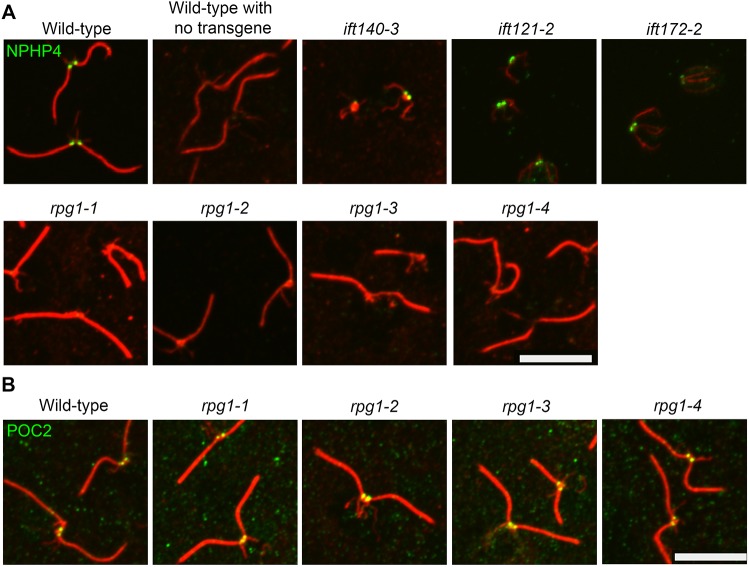
**Localization of NPHP4 and POC2 in newly isolated mutants.** (A) A strain with the *NPHP4* gene tagged with the HA epitope was crossed into each mutant to determine whether NPHP4 is properly localized. Top row, wild type; wild type with no transgene; *ift140-3*; *ift121-2*; *ift172-2.* Bottom row, *rpg1-1*; *rpg1-2*; *rpg1-3*; *rpg1-4*. (B) Wild type and *rpg1* mutants were stained with antibodies against POC2. Scale bars: 10 μm.

We performed an unbiased forward genetic screen for mutants that fail to assemble cilia and a secondary screen of these strains for ones that fail to localize NPHP4 properly. We identified four mutant strains, called IB4, DB35, GB10 and GB24, that lack NPHP4 at the basal bodies by immunofluorescence analysis of cell expressing a *HA::NPHP4* transgene (Awata et al., 2014) (Fig. 1A). Although we isolated the *rpg1* (herein, we use *RPG1* to denote *Chlamydomonas* homolog of mammalian *RPGRIP1L*) mutants in a screen for cells lacking cilia, we found that upon removal of the cell wall, the cells had assembled cilia. The newly divided *rpg1* mutant cells fail to be released from the mother cell wall in a process called hatching. Cells lacking cilia show a similar failure to hatch. These mutant strains have no obvious mitotic or meiotic defect. They were subjected to whole-genome sequencing and were all found to carry mutations in the *RPG1* gene and they were renamed as *rpg1-1* through *rpg1-4* (Table 1). One of the mutants, DB35, carries mutations in both *RPG1* and *IFT121*. Through a backcross of DB35, we obtained progeny (DB35-1) that contained only the *ift121-2* mutation and progeny (DB35-2) that contained only the *rpg1-2* mutation. Both genotypes showed a loss of cilia. To determine whether the *rpg1* mutations are causative for the observed phenotype, we used linkage analysis (Lin and Dutcher, 2015). For each of the four *rpg1* mutants, we found that the lack of cilia co-segregates with the mutation in *RPG1* by PCR (Table 1).

**Table 1.**
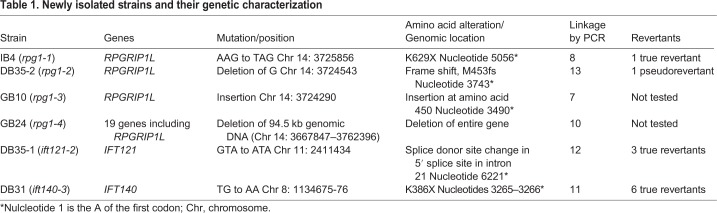
**Newly isolated strains and their genetic characterization**

As a second line of evidence, we used reversion analysis to identify intragenic events that restore function. Since the *rpg1-3* and *rpg1-4* alleles contain either a large insertion or a deletion, respectively, which are highly unlikely to produce intragenic events, we mutagenized the *rpg1-1* and *rpg1-2* strains to isolate revertants/suppressors that restore swimming. We found one true revertant of *rpg1-1* and 1 pseudorevertant (E11a) of *rpg1-2* that deletes 41 nucleotides and restores the predicted reading frame with a 14 amino acid deletion, which we renamed *rpg1-5* (Fig. S1). We conclude that mutations in *RPG1* are causative for the observed phenotype given that we have four independent alleles with the same phenotype (Tulin and Cross, 2015), the phenotype and mutant alleles co-segregate, and we were able to obtain revertants for two different *rpg1* alleles.

Diener and colleagues have previously analyzed isolated transition zones by proteomics and found that POC2 localizes to this region (Diener et al., 2015). To ask whether RPGRIP1L affects the localization of this protein, we used antibodies to POC2. We found that POC2 properly localizes in the *rpg1-1* strain (Fig. 1B).

### Localization of NPHP4 is not affected in other mutants that lack cilia

In our unbiased forward genetic screen for strains lacking cilia, we found mutations in two *IFT* genes. In addition to *ift121-2* in DB35 (Lin et al., 2018), a mutation in *IFT140* (DB31) was also identified. By using the same strategy as above, we found that the *ift140-3* and *ift121-2* mutations show linkage between the mutant allele and the mutant phenotype (Table 1). We found three and six true revertants of the *ift121-2* allele and the *ift140-3* allele, respectively (Table 1). We conclude that the mutations in *IFT140* and *IFT121* are causative for the observed phenotype based on both co-segregation and reversion. In addition, we have previously shown that *ift121-2* is rescued upon expression of the wild-type *IFT121* gene (Lin et al., 2018).

**Fig. 2.**
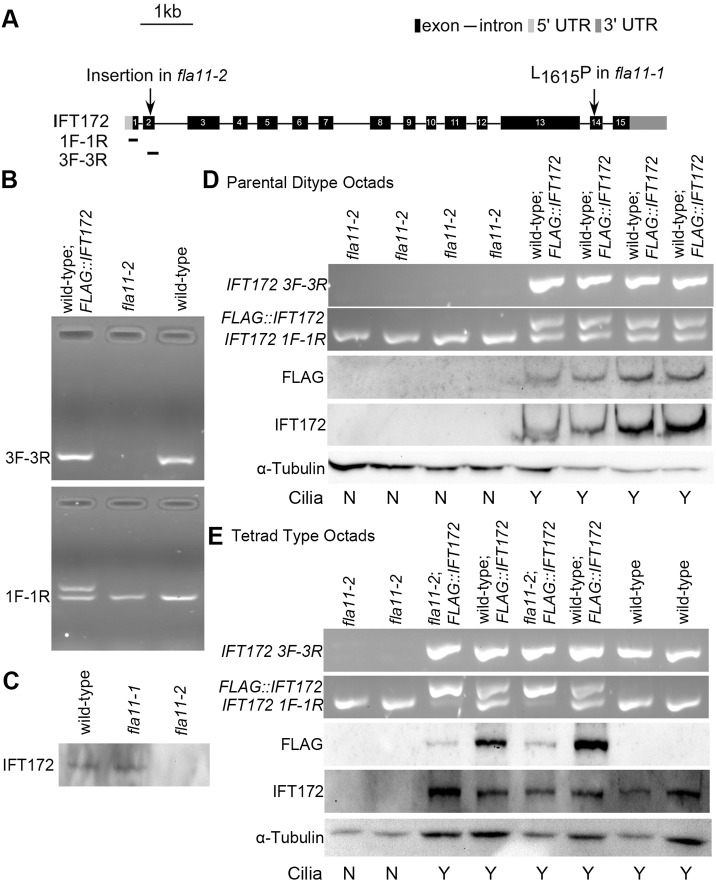
**Characterization of the *fla11-2* mutation in the *IFT172* gene.** (A) Diagram of the gene structure of *IFT172* with the insertion in exon 2. The location of primer sets 1 and 3 used in B are indicated. (B) PCR verification of the insertion with primer set 3 (top panel) and the presence of FLAG-tag with primer set 1 in wild-type strain carrying the transgene (bottom panel). (C) Immunoblot of IFT172 in whole-cell lysates of wild-type, *fla11-1* and *fla11-2* strains. (D,E) Co-segregation of the insertion and rescue by the 3× FLAG::IFT172. In parental ditype and tetrad type octads, the presence of cilia is assessed (N, no cilia; Y, have cilia) and compared to the presence of the insertion (primers, 3F, 3R), the presence of the transgene (primers 1F, 1R) and immunoblots with antibodies to the FLAG tag or IFT172. The level of α-tubulin is shown as a loading control.

It had been previously noted that strain CC-4348 (Goodson et al., 2011) lacked cilia. Here, CC-4348 was backcrossed five times, and two progeny lacking cilia were retained. The mutant phenotype (lack of cilia) maps to a region of about 500 kb on chromosome 17 based on ∼500 meiotic progeny. The two strains were subjected to whole-genome sequencing (Table S2) and we identified an insertion in exon 2 of the *IFT172* gene. Primers (Fig. 2A; Table S3) surrounding the suspected insertion amplify a 212 bp fragment from wild-type but not from the CC-4348-derived strains (Fig. 2B, top panel). This insertion in *IFT172* shows linkage to the mutant phenotype in 62 meiotic progeny from a cross of the CC-4348-derived strain with wild type (CC-125).

In immunoblots using an antibody against IFT172 (Pedersen et al., 2005), we observed IFT172 in the cytoplasm of both wild type and the temperature-sensitive *ift172* mutant, *fla11-1*, at 21°C but not in the sequenced strains (Fig. 2C). A 3× FLAG-tagged *IFT172* gene (Fig. 2B, bottom panel) was introduced into wild-type cells and we isolated a transgenic strain that expressed the FLAG::IFT172 protein with the correct protein size. This transgene was introduced into the CC-4348-derived strains by meiotic crosses. The ciliary assembly defect was rescued; over 90% of the mutant cells with the transgene had cilia (*n*=200). By PCR, we confirmed that the tagged gene co-segregated with rescue in all 18 tetrads analyzed. We selected a parental ditype octad and a tetratype octad to ask whether the tagged protein co-segregates with motility (Fig. 2D,E). The tagged protein is recognized by both anti-FLAG and anti-IFT172 antibodies and the transgene co-segregates with the rescue of ciliary assembly. Thus, we conclude that these strains carry a null mutation in *IFT172*. We named this allele *fla11-2*.

We asked whether *IFT* mutants affect NPHP4 localization at the transition zone. We assayed the localization of NPHP4 in *ift121-2*, *ift140-3*, *fla24-1* (a DHC1b mutant; Lin et al., 2013b), *fla9* (*ift81-2*), *fla11-1* (*ift172-1*), *fla11-2* (*ift172-2*), and rescued *fla11-2* with the rescuing transgene. All of these mutants show NPHP4 staining at the transition zones (Fig. 1A; Fig. S2). Hence, NHPH4 is localized properly in cells lacking cilia and IFT is not needed for NPHP4 localization.

### Assaying the ciliary gate in mutants

The transition zone serves as a gate to regulate the entry of cytoplasmic proteins based on the presence of a limited number of proteins assayed in animal cells (Nachury et al., 2010; Reiter et al., 2012; Williams et al., 2011; Li et al., 2016). The region also serves a gate preventing ciliary proteins from leaving (Li et al., 2016). Since cilia can be easily detached from intact *Chlamydomonas* cells and purified away from the cell bodies by centrifugation, *Chlamydomonas* permits a comprehensive biochemical examination of the role of the transition zone as a gate (Craige et al., 2010; Awata et al., 2014). If the transition zone serves as a gate between the cytoplasm and cilia, the isolated cilia from mutants would be expected to contain proteins that are not normally present in the cilia. If it serves in protein transport or retention, known ciliary proteins would be expected to be absent. Importantly, ciliary excision occurs distal to the transition zone domain, so transition zone proteins are not expected to be in the ciliary proteome (Lewin and Lee, 1985).

We examined the ciliary protein composition of the transition zone mutant *rpg1-1* and of temperature-sensitive mutants with defects in IFT (*fla11-1* and *fla24-1* after 4 h at 32°C) (Table 2). Although the *rpg1-1* mutant was isolated as a strain lacking cilia, cells with cilia are observed when the cell wall is removed with autolysin (Dutcher, 1995). Two independent cilia samples from each of the three strains were isolated. The number of peptides from α-tubulin and β-tubulin is similar among all of the independent preparations in each group (wild type versus mutants with IFT defect, and wild type versus *rpg1-1*, Table S4), which serves an internal comparison of the mass spectrometry data.

**Table 2.**
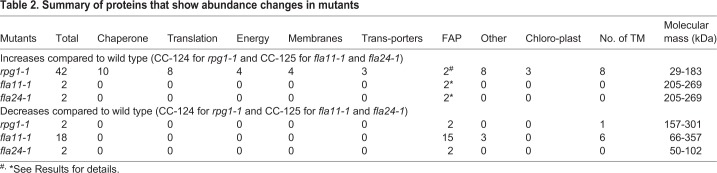
**Summary of proteins that show abundance changes in mutants**

**Fig. 3.**
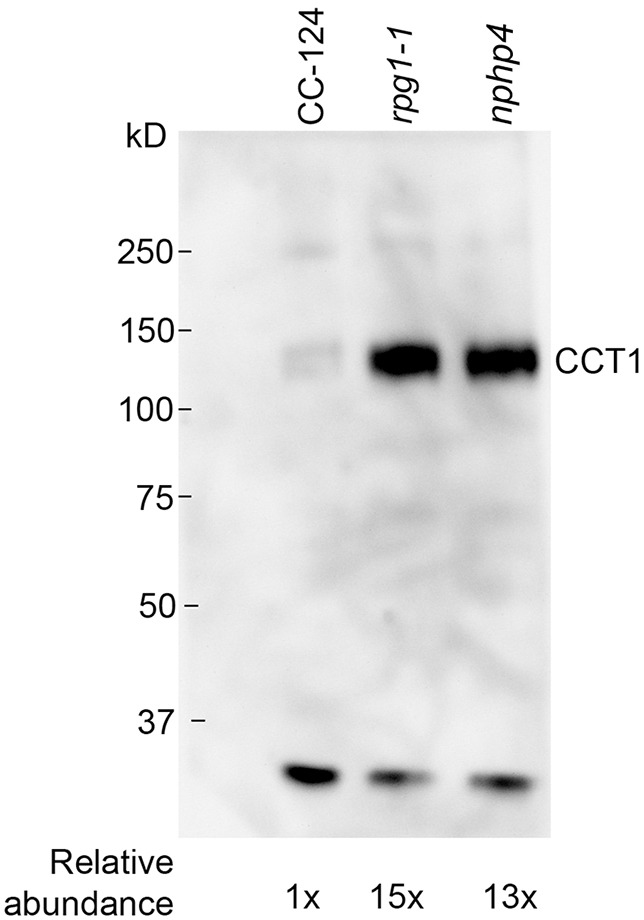
**The chaperonin, CCT1, is increased in *rpg1-1* and *nphp4* cilia.** Ciliary proteins (4 μg) from wild-type, *nphp4*, and *rpg1-1* cells were used. Proteins were separated on a 7.5% acrylamide SDS-PAGE gel. The small cross-reactive protein was used as a loading control.

The most striking increase in nonciliary proteins is observed in the *rpg1-1* mutant. A total of 40 nonciliary proteins were present in the isolated *rpg1-1* cilia and they range in size from 29 to 183 kDa (Table 2). These proteins fall into several categories (Table S5); these are chaperonins (Table S5, yellow), and proteins involved in translation (orange), ATP production (blue), membrane remodeling (red) and transport (purple). The TRiC/CCT chaperonin complex, heat shock protein 70 (HSP70G) and RB60 (a protein disulfide isomerase) are present in these cilia (Table S5). The TRiC/CCT chaperonin proteins facilitate folding of ∼10% of the eukaryotic proteome (Cong et al., 2010; Kalisman and Levitt, 2010; Leitner et al., 2012). All of the components of the CCT/TRiC complex are present in *rpg1-1* cilia*.* We performed immunoblots with antibodies to CCT1 and see an increase of 15-fold in *rpg1-1* cilia compared to wild type (Fig. 3). Since *rpg1-1* cells fail to localize NPHP4, we examined isolated cilia from *nphp4* mutants and see a 13× increase of CCT1 in the cilia compared to wild type by immunoblotting (Fig. 3).

Four translation initiation factors (EIF3A, EIF3B, EIF3C and EIF2G), a tRNA synthetase, an RNA helicase and several other proteins involved in translation are present in the *rpg1-1* cilia (Table S5). This is consistent with results from Craige et al. (2010) and Awata et al. (2014) showing that the amount of elongation factor 3 is increased in both *cep290* and *nphp4* cilia, and four tRNA synthetases are increased in *nphp4* cilia. Four of the cytoplasmic proteins found to show increased levels affect membrane remodeling and vesicle formation. Proteins of the mitochondrial F0/F1 ATP synthase (ATP-2, ASB-1 and ASG-2) and several protein transporters and ion channels are increased. We also observe three chloroplast-localized proteins (Table S5, green). Thus, we do not know whether these represent contamination or these proteins are actively moved from the chloroplast into cilia. Surprisingly, most of the flagellar-associated proteins (FAPs) that show changes in *cep290* and *nphp4* (Awata et al., 2014; Aanstad et al., 2009; Craige et al., 2010) are not identified in our *rpg1-1* dataset.

The main alteration in the two *ift* mutants is the loss or reduction of proteins (Table 2). In *fla11-1* cilia, the levels of 18 proteins are reduced, and two are reduced in *fla24-1* cilia. The missing proteins range in size from 50 to 357 kDa. In *fla11-1* cilia, the levels of IFT-A complex proteins IFT121, IFT122, IFT139, IFT140 and IFT144 are significantly reduced, whereas the sixth IFT-A subunit, IFT43, is not reduced. The levels of IFT-B complex proteins show no reduction in *fla11-1* cilia. The IFT complex proteins are not affected in *fla24-1* cilia (Table S6). Six of the 13 non-IFT proteins that show reduced levels have transmembrane domains. This suggests that IFT172 may play a role in the entry or retention of proteins associated with the ciliary membrane.

We identified changes in our wild-type strains from the proteomics data. Two FAPs, FAP102 and NSG1 (also known as CYN7), are missing in our wild-type strain (CC-124) but are present in another wild-type strain (CC-125) as well as in *rpg1-1* (# in Table 2). Analysis of the sequence for our wild-type strains does not show any SNP or indels in these genes. We do not know the genetic cause. CPC1 and FAP42 (asterisks in Table 2) are missing in our other wild-type strain (CC-125) (Table S6). CPC1 is part of the central pair complex (Zhang and Mitchell, 2004). Using antibodies to CPC1 (Zhang and Mitchell, 2004), we showed that CPC1 is absent from our CC-125 cilia but present in CC-124, *fla11-1* and *fla24-2* cilia by immunoblotting (Fig. S3). By whole-genome sequencing (Lin et al., 2013a), we found that our CC-125 strain had acquired a point mutation that leads to a F31C change that appears to disrupt CPC1 localization to cilia. This change was confirmed by PCR followed by enzyme digestion to distinguish the difference between our CC-125 and CC-124, the other wild-type strain (Fig. S3). The *cpc1* mutation is unique to our strain and is not in the CC-125 stock from the *Chlamydomonas* Resource Center (Fig. S3). Thus, the level of CPC1 is not increased in the mutant cilia due to an IFT defect in *fla11-1* and *fla24-1* but due to a mutation in our CC-125 strain. We refer to this mutation as *cpc1-2*. It is possible that the change in FAP42 is also related to the central pair mutation. Enolase, which interacts with CPC1, is not affected in the *cpc1-2* strain, based on our mass spectrometry results, whereas it is affected in the *cpc1-1* strain (Zhang and Mitchell, 2004; Mitchell et al., 2005).

Our mass spectrometry data from two wild-type strains (CC-124 and CC-125) also allowed us to identify 16 novel proteins that were not previously identified in the *Chlamydomonas* Flagellar Proteome (Pazour et al., 2005) (Table S7). Six of these proteins are not found in the JGI Chlamydomonas version 4 genome sequence and one of them has an incomplete v4 gene model. Eight of the 16 proteins were recently added to the updated Flagellar Proteome (http://chlamyfp.org/). They are indicated in Table S7. One protein, ALA2, which is an aminophospholipid transporter, is found in the transition zone proteome (Diener et al., 2015). The TRP15 protein, a member of the transient receptor potential family, has been localized to *Chlamydomonas* cilia (Fujiu et al., 2011).

## DISCUSSION

Our unbiased forward genetic screen identified four strains (Table 1) that lack NPHP4 at the transition zone from a collection of 250 strains lacking cilia. These four mutants each carry a unique mutant allele of *RPG1*. Given the genetic heterogeneity in worms (Li et al., 2016; Williams et al., 2011) and the interaction network observed in tissue culture cells (Sang et al., 2011; Gupta et al., 2015) at the transition zone, we are surprised that our screen did not reveal additional genes. The *Chlamydomonas nphp4* mutant assembles cilia. The *rpg1* and *cep290* mutants assemble cilia, but fail to release cells from the mother cell wall (Craige et al., 2010). Therefore, other transition zone mutants may assemble cilia, and not be identified in our screen. It is also possible that secondary screens for the failure to localize transition zone proteins other than NPHP4 would identify additional transition zone defective mutants in our collection. The lack of cilia observed in *rpg1* and *cep290* mutants appears to arise from the failure of cells to hatch from the mother cell wall as this phenotype is rescued by removal of the cell wall. We hypothesize that these transition zone proteins may be involved in the production of ectosomes that contain the lysin needed for hatching (Wood et al., 2013).

The use of super-resolution microscopy has revealed that multiple transition zone proteins (NPHP1, TCTN2, TMEM231 and AHI1) fail to localize properly in human fibroblasts that carry *RPGRIP1L* mutations (Shi et al., 2017). This is consistent with our study showing that NPHP4 is missing in the *rpg1-1* mutant, given that NPHP1 and NPHP4 are part of the NPHP module. Analysis of *cep290* and *nphp4* mutants in *Chlamydomonas* (Awata et al., 2014; Craige et al., 2010) suggested a role of these two proteins in preventing entry of inappropriate transmembrane domain proteins. In our analysis of *rpg1-1*, eight of 40 proteins have a transmembrane domain. The set of cytoplasmic proteins range in size from 29 to 190 kDa, which suggests that RPG1 does not have a specific size range for the exclusion of ciliary proteins. Comparison of protein compositions in *rpg1-1* and *nphp4* (Awata et al., 2014) suggest that the *rpg1-1* mutant shows a stronger defect and allows the entry of more nonciliary proteins. Even though the lists of enriched proteins found in *rpg1-1* and *nphp4* do not overlap, by immunoblotting we showed that CCT1, part of the TRiC/CCT chaperonin complex, increases in the cilia of both *rpg1-1* and *nphp4* gametic cells (Fig. 3). We suggest that the lack of overlap may reflect differences in the life cycle of the cells used. Awata et al. used cells growing exponentially, while we used gametic cells. In the future, it will be interesting to test how the activity of the gate varies with the stage of the life cycle.

In *Chlamydomonas*, the mitochondrial ATP synthase and the TRiC/CCT are excluded from wild-type cilia (Table 2; Pazour et al., 2005). In *C. elegans*, the male sensory neurons contain ATP synthase whereas the olfactory AWA sensory neurons do not (Hu and Barr, 2005). Four of the TRiC/CCT subunits are found in *Tetrahymena* cilia (Seixas et al., 2003) and in mammalian motile cilia (Seixas et al., 2003; Lopez-Fanarraga et al., 2007). The TRiC/CCT subunits associate with mammalian-specific BBS proteins (BBS6, BBS10 and BBS12), which are not present in the genome of *Chlamydomonas* (Seo et al., 2010). These differences provide striking examples that the ciliary gate can differ among cilia in the same organism and in different organisms. These examples suggest that ciliary gate is a better label than ciliary barrier as a gate can be opened and closed in different tissues and cells. FOX1 is present in the *rpg1-1* mutant preparation (Table S5), and is in the transition zone proteome (Diener et al., 2015). In addition, it is found in extracellular vesicles or ectosomes (Long et al., 2016). We suggest that it may be a transient protein in the ciliary compartment in wild-type cells but becomes trapped in the *rpg1-1* mutant.

The *fla11-1* and *fla24-1* mutants show similar IFT particle number and velocity defects (Iomini et al., 2001). However, more proteins are missing from the *fla11-1* cilia than from the *fla24-1* cilia. Patients with defects in intraflagellar transport genes show a unique set of symptoms. This syndrome is called Jeune's asphyxiating thoracic dystrophy (JATD) or short rib polydactyly syndrome (SRPS) (Schmidts, 2014); these patients have defects in cartilage and bone development. A subset of the patients with mutations in *CSPP1* show reduced levels of ARL13B and ACIII (Tuz et al., 2014), which suggests a role for the proteins in the proper localization or retention of ciliary proteins. The JATD patients often show nonskeletal symptoms that develop later in childhood; these include kidney, retinal degeneration and liver fibrosis symptoms, as well as symptoms seen in Bardet–Biedl syndrome patients (Bujakowska et al., 2014; Halbritter et al., 2013). Some of these phenotypes are associated with defects in the transition zone (Waters and Beales, 2011). Interestingly, this constellation of later-appearing symptoms is observed in all of the JATD patients with the exception of those with mutations in the dynein heavy chain (Schmidts et al., 2013). We suggest that the difference in the transmembrane ciliary composition between *fla11-1* and *fla24-1* may be key in understanding the appearance of the adolescent phenotypes.

Several studies in mammalian cells have found that restricted cilia entry occurs for proteins in the 40–70 kDa range through experiments using an *in vitro*, permeabilized cell model (Breslow et al., 2013) or by using labeled dextran (Kee et al., 2012). Lin and colleagues, by using a chemically inducible trap, found that proteins up to 7.9 nm could enter the cilia (Lin et al., 2013c). The cytoskeletal protein septin is involved in a diffusion barrier in yeast (Takizawa et al., 2000; Barral et al., 2000). Septins play a role in regulating membrane protein movement in cilia (Hu et al., 2010). Unfortunately, mutants in the homolog in *Chlamydomonas* are not available in order to test its role. The ciliary gate has been compared to the nuclear pore, which allows entry of specific proteins into the nucleus. It has been hypothesized that a subset of the nucleoporins found in nuclear pores also participate in forming the ciliary gate (Takao et al., 2014). Nucleoporins were not found in the *Chlamydomonas* transition zone proteome (Diener et al., 2015), but these preparations are missing part of the transition zone. It will be interesting to ask whether NUP98 is missing in the transition zone mutants, and whether the gate phenotype is a direct effect or an indirect effect of NUP98.

RPGRIP1L is in a unique position based on super-resolution microscopy to act as a ciliary gate (Yang et al., 2015; Shi et al., 2017). The *Chlamydomonas* RPG1 protein has unique properties that make it an ideal candidate for forming a gate; ∼450 amino acids in the last third of the protein are predicted to be an intrinsically disordered region (IDPs). The human RPGRIP1L also contains a disordered region. IDPs have been implicated in creating compartments without membranes via liquid–liquid phase transitions in the cytoplasm (Zhang et al., 2015), in the nucleus (Berry et al., 2015), in P-bodies in *C. elegans* (Brangwynne et al., 2009), in the pericentriolar material of the centrosome (Woodruff et al., 2015), as regulators of post-translational modifications (Das et al., 2016) and as platforms for transcriptional activation (Kwon et al., 2013). RPGRIP1L clearly plays a role in multiple organisms as part of a transition zone compartment that functions to block entry of proteins from the cytoplasm to the cilium and as a membrane diffusion barrier (Jensen et al., 2015; Williams et al., 2011; Huang et al., 2011). Modulation of the ciliary gate by changes in the life cycle will be an interesting new area to pursue.

## MATERIALS AND METHODS

### Strains and culture conditions

Strain CC-4348 was backcrossed to wild-type cells five times to remove the *arg7-7*, *sta6*, and *cw15* mutations, as well as several mutations that affect ciliary assembly and function that have not been identified to date. The CC-125, CC-1920 (*fla11*-*1*), CC-3866 (*fla24-1*), CC-4348 (*sta6-1*), CC-5116 (*HA::NPHP4*), and CC-1918 (*fla9*) strains were obtained from *Chlamydomonas* Resource Center. The CC-124 and *cpc-1-2* strains were maintained as in-house stocks. The tagged protein (HA::NPHP4) was introduced into various mutant strains by meiotic crosses and detected by PCR. All mutants and epitope tags were verified by PCR (Table S3).

### Molecular mapping and whole-genome sequencing

Progeny of CC-4338 lacking cilia with an intact cell wall were crossed to the highly polymorphic strain CC-1952 (S1C5), and 500 progeny was used in mapping. Crude DNA preparation, PCR and digestion of individual progeny for meiotic mapping were performed as previously described (Lin and Dutcher, 2015). No recombinants were found with the primers at 0.57 Mb and 1.09 Mb. Two independent progeny lacking cilia (4348-1 and 4348-2) were subjected to whole-genome sequencing (Lin and Dutcher, 2015). We used Softsearch (Hart et al., 2013) to find breakpoints, which would indicate >200 bp insertions/deletions, within the mapped region of chromosome 17. In the 4348-1 strain, 379 breakpoints were identified; in the 4348-2 strain, 272 breakpoints were identified. Common breakpoints found in both strains were compared to 292 breakpoints found in a wild-type strain (CC-124) in the same interval.

### Plasmid DNA constructs and *Chlamydomonas* transformation

The BAC DNA 4P11 (*Chlamydomonas* Resource Center), was digested with *Xma*I and *Hind*III. A 10.9 kb fragment, which contains the full-length *IFT172* gene, was gel-purified and ligated into a pBlueScript vector (Stratagene) digested with the same enzymes to form the pBS-*IFT172* plasmid. The 3× FLAG tag was inserted immediately after the start ATG codon via nested PCR. In the first round of PCR, three different PCR products were generated. The first PCR product, a 474 bp fragment, was amplified by primers *Hind*III-F and IFT172-start-R (see Table S3 for primer sequences) with pBS-*IFT172* as a template. The second PCR product, a 95 bp fragment that contains the 3× FLAG tag, was amplified by primers IFT172-start-FLAG-F and IFT172-start-FLAG-R with pBS-3×FLAG (Lin and Goodenough, 2007) as a template. The third PCR product, a 329 bp fragment, was amplified by primers start-FLAG and *Bsm*I-R with pBS-*IFT172* as a template. In the second round of PCR, the 474 bp fragment and the 95 bp fragment were combined as templates. A 550 bp fragment was amplified with primers IFT172-*Hind*III-F and IFT172-start-FLAG-R. In the third round of PCR, the 550 bp fragment from the second round and the 329 bp fragment from the first round were combined as templates. Primers IFT172-*Hind*III-F and IFT172-*Bsm*I-R were used to generate an 863 bp fragment, which was digested with *Hind*III and *Bsm*I, and cloned into pBS-*IFT172* digested with the same enzymes. The resultant plasmid pBS-*FLAG*-*IFT172* was subjected to Sanger sequencing to verify the sequence. *Chlamydomonas* transformation was performed as previously described (Lin et al., 2013a). Wild-type cells (CC-125), were transformed with 1 µg of pBS-*FLAG*-*IFT172* and 1 µg of pSI103, which confers resistance to paromomycin (Sizova et al., 2001); 80 paromomycin-resistant colonies were obtained. Seven FLAG-positive colonies were identified using PCR. These were subjected to immunoblotting with an anti-FLAG antibody. One positive strain, which contains expressed FLAG–IFT172 proteins, was identified and subsequently crossed to the *fla11-2* and *fla11-1* mutants*.*

### Immunoblotting and immunofluorescence

SDS-PAGE and immunoblotting were performed as previously described (Lin et al., 2013a). Primary antibodies used in this study were: anti-FLAG antibody (Lin and Goodenough, 2007), M2 monoclonal, Sigma-Aldrich (F2555, SIG1-25, 1:2000 dilution), anti-IFT172.1 antibody (Pedersen et al., 2005, 1:200 dilution), anti-CPC1 antibody (Zhang and Mitchell, 2004, 1:100 dilution), anti-CCT1 antibody (Sigma-Aldrich, HPA027337, 1:200 dilution), and anti-α-tubulin antibody (Sigma-Aldrich, T6199, lot number 091M4813, 1:2000 dilution). Secondary antibodies include horseradish peroxidase (HRP)-conjugated goat anti-mouse-IgG antibody (BioRad, 1721011, 1:5000 dilution) and HRP-conjugated goat anti-rabbit-IgG antibody (Sigma-Aldrich, A6154, 1:5000 dilution). For immunofluorescence, cells were treated with autolysin for 30 min to remove cell walls. Cells were allowed to adhere to poly-L-lysine-coated glass slides for 2 min at room temperature in the dark, lysed with 1% Nonidet P-40 for 2 min, fixed with 4% paraformaldehyde for 15 min at room temperature and then subjected to fixation in cold methanol for 5 min at −20°C. Primary antibodies used include anti-HA High Affinity antibody (3F10, Roche, 11867423001, lot number 13500600, 1:200 dilution) and anti-acetylated-α-tubulin (Sigma-Aldrich, T7451, lot number 036M4856V, 1:250 dilution). Secondary antibodies used include Alexa Fluor 488-conjugated goat anti-rat-IgG antibody (Invitrogen API83P, lot number 2915317, 1:500 dilution) and Alexa Fluor 594-conjugated chicken anti-mouse-IgG antibody (Invitrogen, A21201, lot number 737674, 1:500 dilution).

### Cilia isolation, fractionation, silver staining, mass spectrometry and data analysis

The *rpg1* cells were treated with autolysin to release cells from the mother cell wall before cilia isolation (Craige et al., 2010; Kubo et al., 2015). Cilia were detached from cell bodies by pH shock and isolated as previously described (Lin et al., 2013b). Isolated cilia were resuspended in HMDEK buffer (Awata et al., 2014). Proteins (5 μg) from each sample were subjected to in-solution tryptic digestion and mass spectrometry (Donald Danforth Plant Science Center, St Louis, MO).

We used the following criteria to construct the lists of proteins that changed in the mutant compared to wild-type preparations: (1) for a protein to be considered as increased in the mutant, it must have greater or equal to 10 peptides on average from two biological replicates while it has no peptides in the wild-type preparation, and (2) for a protein to be considered as decreased in the mutant, it must show greater than a five-fold reduction compared to the number of peptides found in wild-type cilia and there must be greater than or equal 10 peptides in the wild-type preparation.

The Phytozome *Chlamydomona*s v5.5 accession numbers of proteins that have an average number of five or more peptides in all four wild-type (CC-124 and CC-125) samples were converted into JGI Chlamydomonas v4 protein IDs from the file provided by Phytozome and compared to the Flagellar Proteome JGI v4 IDs. Sequences of proteins not found in the Flagellar Proteome were subjected to BLAST analysis against the v4 genome sequences to query whether the proteins were predicted in the v4 genome. Some proteins do not have a match in the v4 genome. Some proteins have v4 protein IDs but are not found in the flagellar proteome. These proteins are considered novel ciliary-associated proteins.

### Isolation of mutants lacking cilia

Wild-type cells (∼5×10^8^) were mutagenized with ultraviolet light (750 μJ) and resuspended in 48 25×150 mm culture tubes at 25°C. Cells at the bottom of the tubes were transferred into 20 ml of fresh medium after 2–3 days. This was repeated six times. Single colonies were obtained by plating ∼100 cells onto solid medium from cultures with cells found primarily at the bottom of the culture tubes. Single colonies from ∼250 independent mutant cultures were examined for the presence or absence of the HA-tagged NPHP4 protein at the basal bodies by immunofluorescence.

## Supplementary Material

Supplementary information
