# Supplementary material for: RPGRIP1L helps to establish the ciliary gate for entry of proteins
Source: J Cell Sci. 2018 Oct 26;131(20):jcs220905. doi: 10.1242/jcs.220905 (PMC6215392; doi:10.1242/jcs.220905)
Supplement: Supplementary information [file joces-131-220905-s1.pdf]

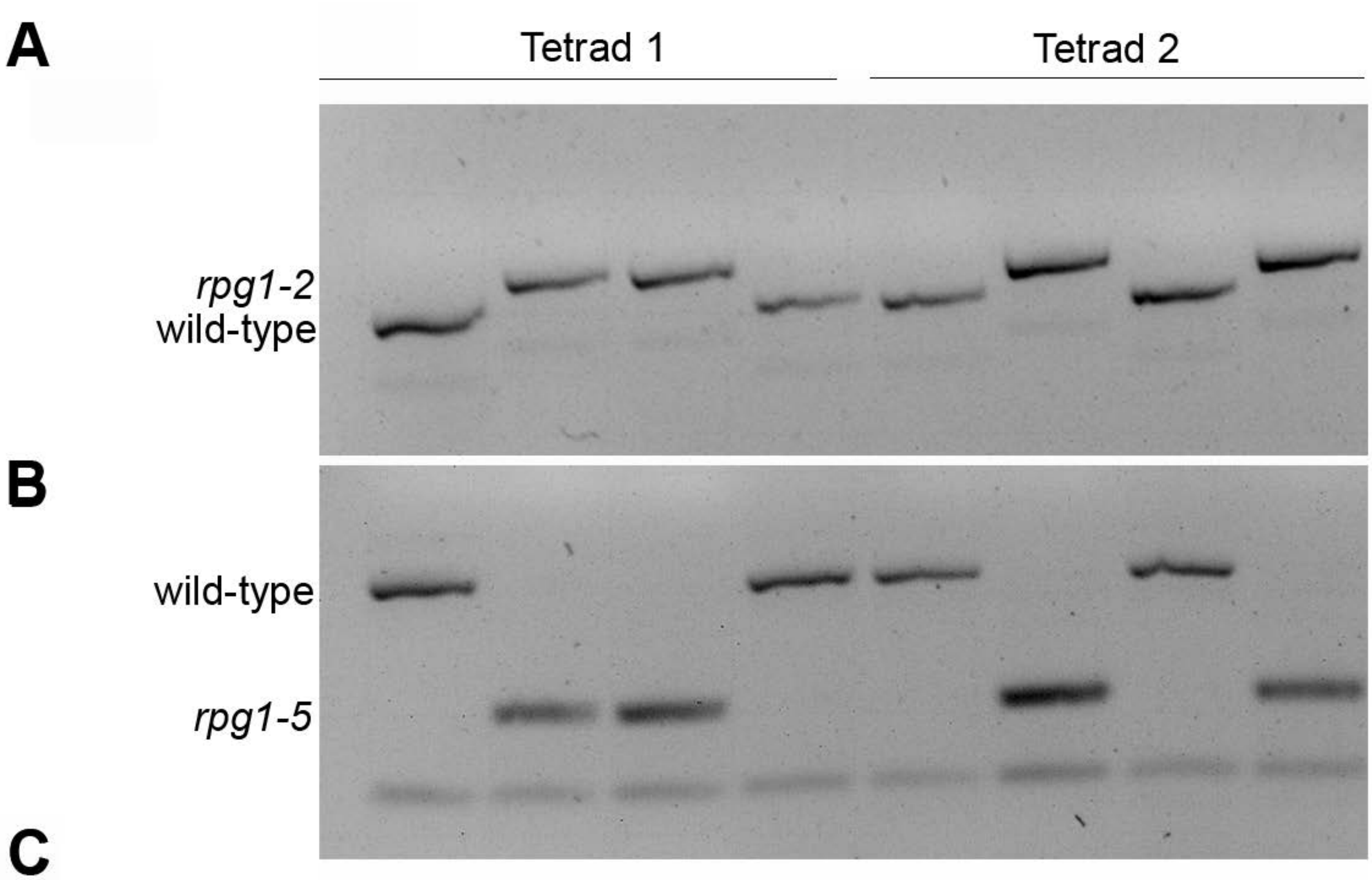

C

```
EVKASFDELDGDRDGCLTHLEVVRLVRRFLKDITRRRCATCWPSCSSGTCWARAASALTCSTS   rpg1-2
|||||||||||||||||||||||||||||||
EVKASFDELDGDRDGCLTHLEVVRLVRRFLKDMYQKEVRYLLAKLQQWDVLGESRVSFDELYQ   wild-type
||||||||||||||                   ||||||||||||||||||||||||||||||
EVKASFDELDGDRD--------------LPQGHYQKEVRYLLAKLQQWDVLGESRVSFDELYQ   rpg1-5

AWSWCGCSEWARGWAPPCAPARPCARPHAPPRPARCPPPAPTSAPAAPPARRX             rpg1-2

CLELVRVFRVGQGLGTAMRTGSPMRSPTRSTSPSKVPTAGPNIRASGPARASMIRSTVGGGGM   wild-type
|||||||||||||||||||||||||||||||||||||||||||||||||||||||||||||||
CLELVRVFRVGQGLGTAMRTGSPMRSPTRSTSPSKVPTAGPNIRASGPARASMIRSTVGGGGM   rpg1-5
```

Fig. S1. E11a is a pseudorevertant of *rpg1-2*

The E11a strain (*rpg1-5*) behaves as an intragenic revertant based on crosses to wild-type. In 6 tetrads, the original ciliary phenotype is not recovered. **(A).** The original *rpg1-2* allele is present by PCR from 2 tetrads. The *rpg1-2* mutation leads to the removal of a *Nla*III restriction enzyme site in the PCR product. Thus, the wild-type allele produces a shorter band (133 bp) than the *rpg1-2* allele (219 bp). **(B).** Sanger sequencing revealed a 41 bp deletion in the exon 5' to the original mutation. The wild-type allele results in 239 bp product and the *rpg1-5* allele results in 198 bp product with the E11a primers (Supplemental Table 3). **(C).** The predicted protein sequence for wild-type, *rpg1-2*, and *rpg1-5*.

Journal of Cell Science • Supplementary information

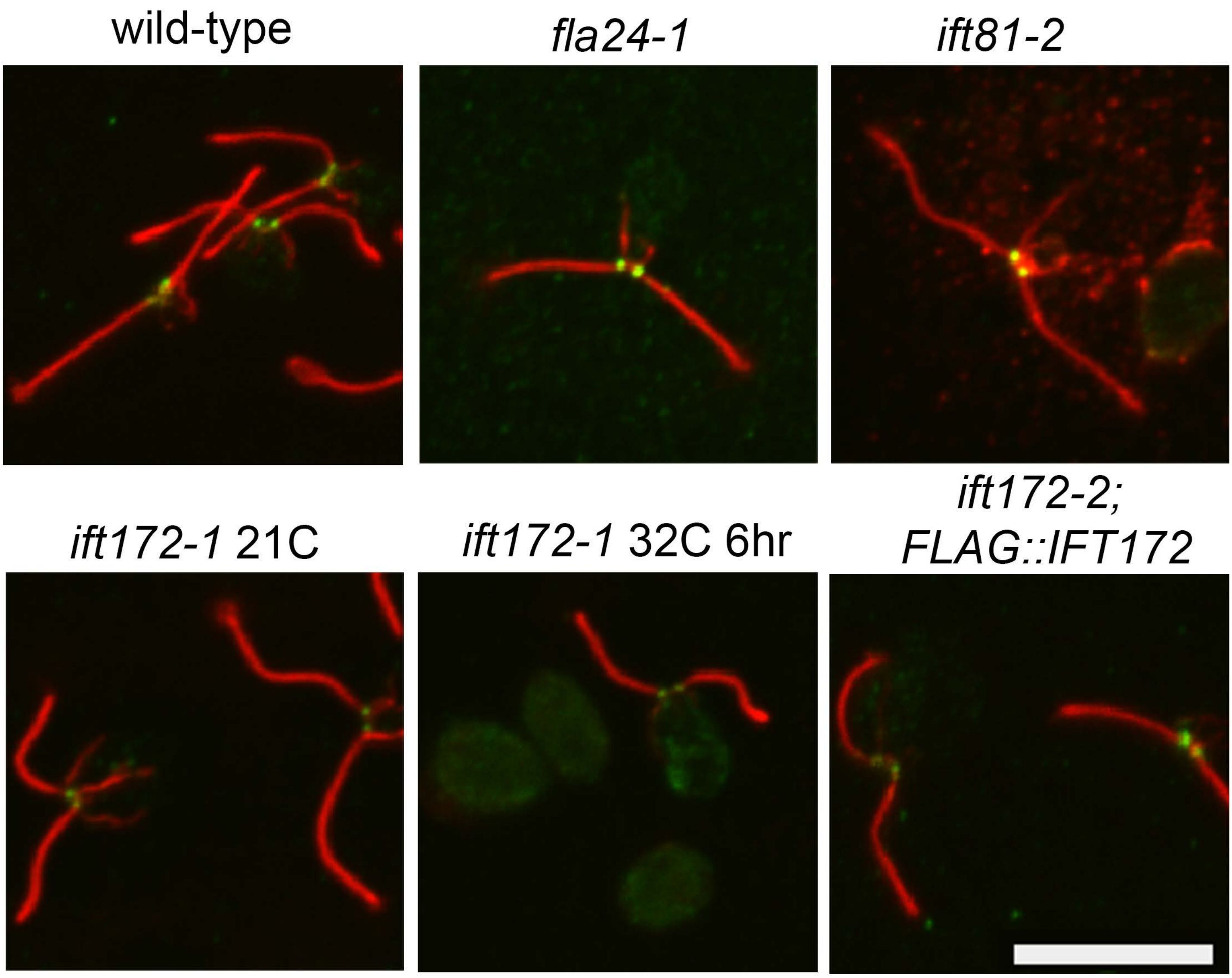

Fig. S2. The transition zone protein NPHP4 shows wild-type localization in *ift* mutant by immunofluorescence. Wild-type, *ift81-2, fla24-1, ift172-1* at permissive and restrictive temperatures, and *ift172-2* with the transgene were stained with antibodies to the HA::NPHP4 transgene. The *ift* mutants are indistinguishable from wild-type cells.

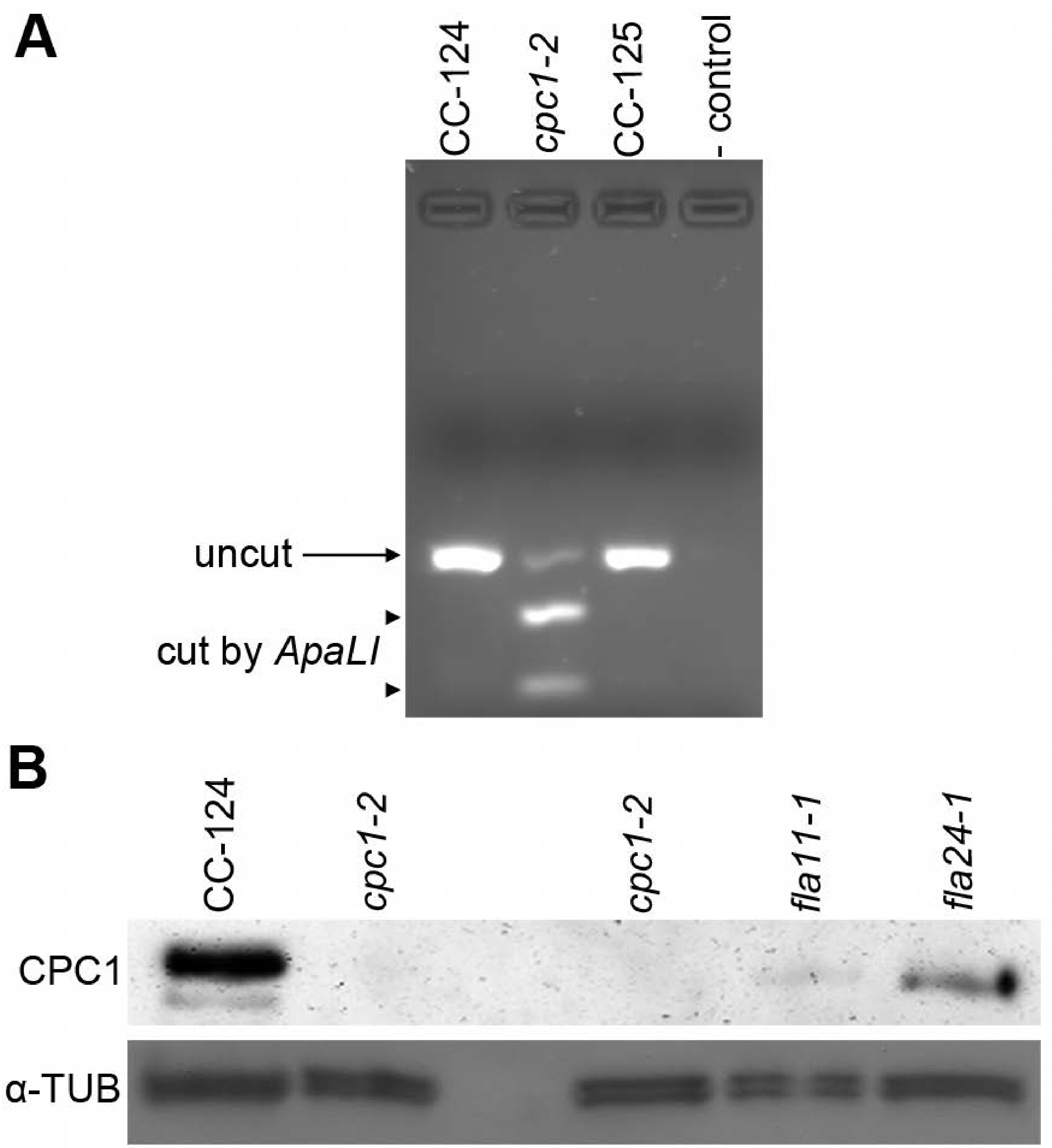

Fig. S3. The mutation in *CPC1* leads to its failure to enter the cilia. **(A)**. The*CPC1* gene was amplified by PCR from CC-124, CC-125 from the *Chlamydomonas* Resource Center, and CC-125 from our lab, which is now renamed *cpc1-2*. The change in *cpc1-2* leads to an enzyme digestion by *Apa*LI. "- control" indicates a PCR sample with no DNA template. **(B)**. Anti-CPC1 antibodies recognizes the CPC1 protein from cilia of CC-124, *fla11*, and *fla24*, but not *cpc1-2*. The immunoblot with an antibody to α-tubulin serves as a loading control.

Journal of Cell Science • Supplementary information

Table S1. Gene names for proteins found in the transition zone

HGNC Names*	Other names in humans	Names in *Chlamydomonas*	Names in *C. elegans*
RPGRIP1L	*NPHP8, MKS5, JBTS7*	*RPG1*	*mks-5*
**MKS Module**			
MKS1	*JBTS28, BBS13*	*MKS1/POC12*	*mks-1*
B9D1	*MKSR1, JBTS27*	*B9D1*	*mksr-1/ mksr-2*
B9D2	*MKSR2, JBTS34*	*B9D2*	
TMEM216	*MKS2, JBTS2*	*TMEM216*	*mks-2*
TMEM67	*MKS3, JBTS6, NPHP11*	*TMEM67*	*mks-3*
CC2D2A	*MKS6, JBTS9*	*CCD2DA*	*mks-6*
TCTN1	*JBTS13*	*TCT1*	*tctn-1*
TCTN2	*JBTS24*		
AHI1	*JBTS3*	*AHI1*	No homolog
TMEM231	*OFD3, TMEM231 MKS11, JBTS20*	*TMEM231*	*tmem-231*
**NPHP Module**			
NPHP1		*NPHP1*	*nphp-1*
NPHP4		*NPHP4/POC10*	*nphp-4*
**CEP290 Module**			
CEP290	*NPHP6, MKS4, JBTS5*	*CEP290/POC3*	*ccep-290*

*HUGO Gene Nomenclature

Journal of Cell Science • Supplementary information

Table S2. Summary of whole genome sequencing results

Strains	Index	Total reads	Aligned reads	Unique aligned reads	Coverage	% Aligned	% Unique aligned	# of SNPs/ indels	unique SNPs/ indels	Gene
4348-1	CCATCAT	62738156	58690723	51532689	49.4	93.5	82.1	83559	1846	*IFT172*
4348-2	TAACAAG	52968064	50062722	43959958	42.1	94.5	83.0	80691	2104	*IFT172*
DB31	GAGGCGT	45448192	38841747	37916934	32.7	85.5	83.4	41672	4412	*IFT140*
DB35	CGTCGCT	43153902	37130207	36254952	31.3	86.0	84.0	42347	4873	*IFT121, RPGRIP1L*
GB10	GTCTGAT	56321380	50055327	48770886	42.1	88.9	86.6	88713	13777	*RPGRIP1L*
GB24	CAAGCCG	55976158	50894225	49593614	42.8	90.9	88.6	89282	13679	*RPGRIP1L*
IB4	GCAGGGG	56569234	50084507	48780868	42.2	88.5	86.2	88868	13612	*RPGRIP1L*

Table S3. Primers used in this study.

Click here to Download Table S3

Table S4: The abundance of TUA1, TUB1 as internal controls

Accession Number	Anno-tation	MW (kDa)	CC-125 average	*fla11-1* average	*fla24-1* average	CC-124 average	*rpg1-1* average	Flagellar proteome	Transition zone proteome	# of TM
Cre03.g190950 .t1.2	TUA1; α tubulin 1	50	48	45	45.5	28	27.5	Y	Y	0
Cre12.g542250 .t1.1	TUB1; β tubulin 1	50	46.5	44.5	44	30.5	28	Y	Y	0

Journal of Cell Science • Supplementary information

Table S5. List of proteins that showed significant changes in abundance in *rpg1-1* cilia compared to wild-type cilia

Click here to Download Table S5

Table S6. List of proteins that showed significant changes in abundance in *fla11-1* and *fla24-1* flagella when compared to wild-type

Click here to Download Table S6

Table S7. List of novel cilia associated proteins

Click here to Download Table S7

Journal of Cell Science • Supplementary information
